# Time-restricted feeding improves metabolic and endocrine profiles in mice with polycystic ovary syndrome

**DOI:** 10.3389/fendo.2022.1057376

**Published:** 2022-12-16

**Authors:** Yan Han, Baiwei Lin, Wenjing Lu, Xu Wang, Wenshuai Tang, Xinge Tao, Han Cai, Chunmei He, Changqin Liu

**Affiliations:** ^1^ School of Medicine, Xiamen University, Xiamen, China; ^2^ School of Life Science, Anhui Medical University, Hefei, China; ^3^ Shanghai Key Laboratory of Metabolic Remodeling and Disease, Institute of Metabolism and Integrative Biology, Zhongshan Hospital, Fudan University, Shanghai, China; ^4^ Fujian Provincial Key Laboratory of Reproductive Health Research, School of Medicine, Xiamen University, Xiamen, China; ^5^ Department of Endocrinology and Diabetes, the First Affiliated Hospital of Xiamen University, School of medicine, Xiamen University, Xiamen, China; ^6^ Fujian Province Key Laboratory of Diabetes Translational Medicine, The First Affiliated Hospital of Xiamen University, Xiamen, China

**Keywords:** time-restricted feeding (TRF), glycolipid metabolism, polycystic ovary syndrome, dietary intervention, endocrine profiles

## Abstract

**Objectives:**

Polycystic ovary syndrome (PCOS) is one of the most common endocrinopathy disorders in premenopausal women, which is characterized by hyperandrogenemia, anovulation, and polycystic ovarian morphology (PCOM). Time-restricted feeding (TRF) is a new intermittent restriction dietary pattern, which has been shown to have positive benefits on obesity and glycolipid metabolism disorders. We aimed to explore the effect of the feeding regimen (ad libitum vs. TRF) on the glycolipid metabolism and reproductive endocrine disorders in a PCOS mouse model.

**Methods:**

PCOS mouse model was induced by continuous subcutaneous administration of dihydrotestosterone for 21 days. Mice were fed a high-fat diet (HFD) for 8 weeks on an ad libitum or time- restricted diet (from 10:30 p.m. to 6:30 a.m.).

**Results:**

Compared to control mice, PCOS mice that received TRF treatment had significantly lower body weight, reduced adiposity, lower area under the curve (AUC) of glucose response in the oral glucose tolerance test (OGTT), and lower AUC in the insulin tolerance test (ITT). TRF also ameliorated lipid metabolism, as shown by a reduction in plasma lipid profiles (triglycerides and cholesterol) and the triglyceride content in the liver of PCOS mice. In terms of reproduction, the plasma androgen level, plasma estrogen (E2) level, and luteinizing hormone (LH)/follicle stimulating hormone (FSH) ratio in PCOS mice were significantly reduced after 8 weeks of TRF treatment. In addition, ovarian histology showed that TRF inhibits cyst formation and promotes corpus luteum formation.

**Conclusion:**

In conclusion, TRF improved metabolic and endocrine profiles in mice with PCOS.

## 1 Introduction

Polycystic ovary syndrome (PCOS) is one of the most common endocrinopathy disorders in premenopausal women. Nearly 8%–13% of women worldwide have been diagnosed with PCOS according to the clinical definition (1). PCOS is a heterogeneous disease characterized by the signs and symptoms of androgen excess, ovarian morphological abnormalities, and dysfunction. Furthermore, patients with PCOS also suffer from comorbidities such as obesity, type 2 diabetes mellitus (T2DM), dyslipidemia, and cardiovascular disease (2), with obesity being one of the most common comorbidities. A large meta-analysis showed that women with PCOS were four times more likely to develop obesity than controls (3). In turn, being overweight or obese can exacerbate hyperinsulinemia further aggravating insulin resistance (IR) in patients with PCOS (4). Researchers also found that dyslipidemia and IR induced by obesity play an important role in hyperandrogenism, which forms a vicious cycle and further aggravates clinical characteristics (5–7).

Treatments for PCOS patients include lifestyle interventions and medical interventions that aim to alleviate clinical symptoms rather than rescue them from pathogenesis, which is still unclear and needs to be further explored. For medical intervention, hormonal contraceptives (HCs) are commonly used to treat menstrual disorders, hirsutism, and acne (8, 9). Off-label administered insulin sensitizers such as metformin and thiazolidinediones (TZDs) have been reported to be effective in the treatment of irregular cycles and hyperandrogenism (10, 11). A recognized successful intervention strategy for weight management is lifestyle treatment including eating pattern modification and increased physical activity (12). Among various therapeutic measures, dietary control is a common intervention to reduce excessive calorie intake and restore circadian rhythm, especially time-restricted feeding (TRF), which is becoming an irreplaceable dietary pattern (13).

TRF is a new intermittent restriction dietary pattern, which suggests that food is available only during the active phase without altering the nutritional composition of food. The defined period can vary from 8 to 12 h, which is enough to ensure sufficient calorie intake compared to *ad libitum* feeding (14, 15). Compared to non-restricted diets, TRF has great advantages in preventing obesity, lowering blood pressure, and improving glucose intolerance in a high-fat diet (HFD)–induced obesity mouse model without changing daily caloric intake between TRF-treated and *ad libitum*–fed mice (16). Previous studies have shown that these benefits of TRF intervention were achieved through improvements in metabolic regulatory mechanisms and the rebuilding of the circadian rhythm (17–19). Moreover, a recent clinical study found that orthodox fasting combined with TRF features could improve blood adiponectin levels in women (20). TRF could also help female mice escape estrous cycle disorders and ovarian follicle dysfunction fed by a chow diet or HFD (21). Anovulatory PCOS patients with TRF also showed substantial improvements in weight loss, hyperandrogenemia, menstruation, and IR (22), which showed good consistency in animal experiments. In a word, numerous experiments have been done to demonstrate the contribution of TRF to metabolic disorders, but little attention has been paid to its effects on PCOS. How TRF affects the reproductive and metabolic aspects of PCOS patients and how it attenuates clinical symptoms in patients with PCOS remained largely unknown. The current study aims to investigate the effect of TRF on the metabolic and endocrine status in mice with PCOS.

## 2 Material and methods

### 2.1 Animals and experiment design

In this study, all mice were of C57BL/6J background and were housed in the Xiamen University Animal Center under a 12-h light/dark cycle in an specific pathogen free (SPF) animal facility under a conventional environment (22°C–24°C and 60%–70% relative humidity). All animal experiments involved were approved by the Institutional Animal Care and Use Committee of Xiamen University School of Medicine.

**Figure 1:**
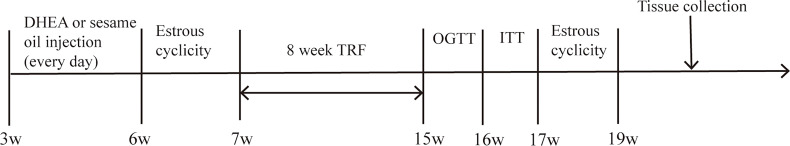
Schematic of the experimental design.

Animals (n = 24) were randomly divided into four groups, and all animals were put on an HFD (60% kcal fat, 20% kcal carbohydrate, and 20% kcal protein; D12492, Research Diets). In this study, mice with a TRF dietary pattern were limited to eating for 8 h (from 10:30 p.m. to 6:30 a.m.) and drinking water *ad libitum*. The TRF model of night eating and day fasting in mice can more accurately mimic human rhythms as mice are nocturnal rhythm animals. For the establishment of the PCOS mouse model, dehydroisoandrosterone (DHEA) has been used to induce related phenotypes (23), while the control was injected with the same volume of sesame oil. The protocol of the study is set in **Figure 1**.

**HFD and*ad libitum* feeding (HA) group** (n = 6). Mice were fed an HFD with *ad libitum* food and water intake, and 100 μl of sesame oil were injected subcutaneously between the scapulae daily for 21 days, starting at 3 weeks of age.

**HFD and TRF intervention (HT) group** (n = 6). Mice were given an HFD as well as a TRF dietary pattern, and 100 μl of sesame oil were daily injected subcutaneously interscapular for 21 consecutive days.

**DHEA and *ad libitum* feeding(DA) group** (n = 6). Mice in this group were given an HFD and water *ad libitum*. When they were 3 weeks old, DHEA (Sigma-Aldrich, #252805; 6 mg/100 g body weight) was mixed in 10 μl 95% ethanol with 90 μl sesame oil and injected subcutaneously daily for 21 days as previously applied (24).

**DHEA and TRF intervention (DT) group** (n = 6). In this group, mice were given an HFD as well as a TRF dietary pattern with daily subcutaneous injections of DHEA (6 mg/100 g body weight) in 10 μl 95% ethanol mixed with 90 μl sesame oil for 21 days.

Thus, TRF groups included the HT group and DT group and non-TRF groups included the HA group and DA group. Body weights were measured weekly at the same time. After a subcutaneous injection of DHEA, reproductive features were evaluated by the estrus cycle, plasma steroid hormone concentration, and ovarian morphology. Glycometabolic features were assessed by the oral glucose tolerance test (OGTT), insulin tolerance test (ITT), lipid profiles in plasma and liver, and liver morphology. The TRF dietary pattern had been administered continuously for 8 weeks, and then, changes in reproductive and glycometabolic features were explored. At the end of the study, tissue and blood samples were collected from fasted mice in the diestrus phase, and plasma was collected by eyeball blood collection and immediately mixed with 7 μl of Ethylene Diamine Tetraacetic Acid (EDTA) on ice. Ovaries and livers were sampled in turn, some of which were fixed in 4% paraformaldehyde, while the rest were stored at -80°C for further experimental procedures.

### 2.2 Estrus cycle

Vaginal smears were used to determine the estrus cycle of mice (25). The vagina of mice was washed with 20 μl of saline, and then, the vaginal lavages were placed on the glass slide. After drying was completed, vaginal cytology was stained with hematoxylin and eosin (H&E) (Biosharp, BL702B, BL703B) and then analyzed under a positive microscope (Leica DM4B, Germany) to determine the specific date of the estrous cycle (26). Vaginal smears were performed daily at 9:00 a.m. on all mice and analyzed for estrous cycles for seven consecutive days.

### 2.3 OGTT and ITT

After overnight fasting (from 5:00 p.m. to 9:00 a.m.), mice were weighed and glucose (2 g/kg body weight) was administered orally by gavage. Blood samples were collected from the tail vein at 0, 15, 30, 60, 90, and 120 min after glucose administration (27, 28). Data were shown as the absolute values of blood glucose concentrations. The total area under the curve (AUC) of glucose response was calculated by GraphPad Prism 5.0 software.

ITT was performed for the mice after fasting for 6 h, 1 week after the OGTT experiment. Mice were injected intraperitoneally with insulin (0.5 U/kg body weight), and blood glucose was monitored at 0, 15, 30, 60, 90, and 120 min after the injection of insulin (29). The AUC was calculated using GraphPad Prism 5.0 software.

### 2.4 Biochemical analysis of steroid hormones and lipid profiles

All blood samples thoroughly mixed with 7 μl of Ethylene Diamine Tetraacetic Acid (EDTA) were centrifuged at 5,000 rpm for 10 min at 4°C; then, the supernatant was carefully collected and stored as a 50 μl tube at -80°C for subsequent hormone evaluation. The plasma concentrations of testosterone, estradiol (E2), follicle-stimulating hormone (FSH), luteinizing hormone (LH), triglyceride, cholesterol, anti-Müllerian hormone (AMH), and leptin were analyzed using commercial ELISA kits (testosterone, Jiangsu Meibiao Biological Technology Co., Ltd, Yancheng, China, MB3306A; E2, Jiangsu Meibiao Biological Technology Co., Ltd, China; FSH, Jianglai Biotechnology Co., Ltd, Shanghai, China, JL13439; LH, Maiman, Jiangsu Feiya Biological Technology Co. Ltd, MM-0582M1; triglyceride, Wako, 290-63701; cholesterol, Wako Lab Assay™ Cholesterol kit; leptin, Crystal Chem, 90030; AMH, Maiman, Jiangsu Feiya Biological Technology Co. Ltd, MM-44204M1).

### 2.5 Liver morphology and ovarian morphology

After collecting liver and ovaries from PCOS mice in the diestrus phase, liver segments at the same site and one ovary in each mouse were excised and then immersed in 4% paraformaldehyde for fixation, gradient dehydration, and embedded with paraffin wax. Sections of 5 µm thickness were collected every 40 µm on slides using a sectioning machine (Leica RM2235, America) according to the conventional histological protocol and were then stained with H&E (Biosharp, BL702B, BL703B).

### 2.6 Statistical analyses

The data were shown as mean ± SEM. One-way ANOVA was performed and followed by *post hoc* comparisons corrected using Bonferroni’s test. All data were analyzed using the statistical software SPSS (version 19.0), and the level of significance was set at p < 0.05.

## 3 Result

### 3.1 TRF prevents weight gain and reduces body fat mass in mice

**Figure 2:**
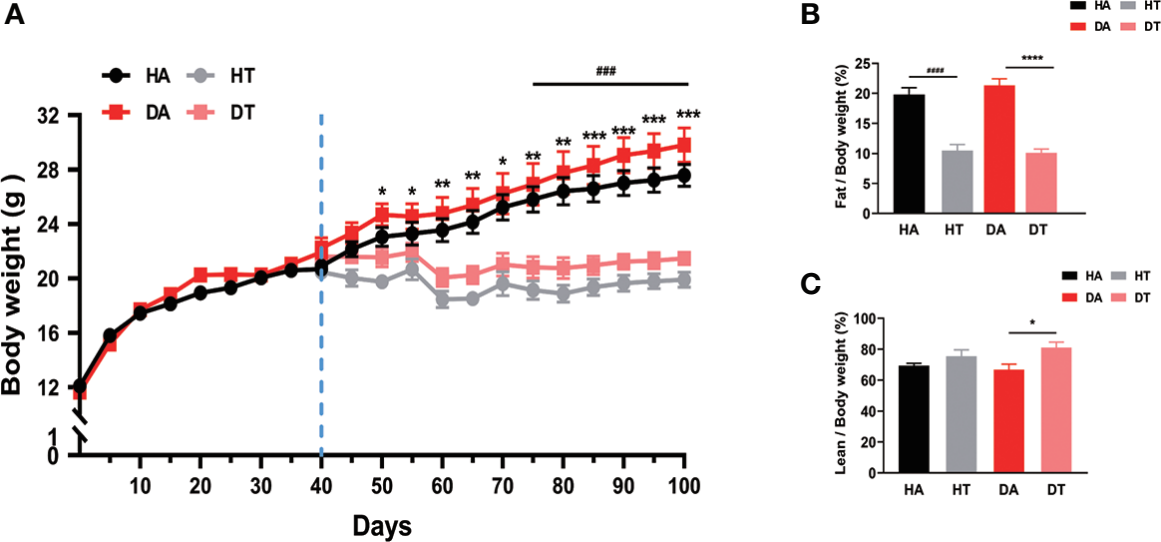
Time-restricted feeding (TRF) prevents weight gain and reduces body fat mass in mice. Growth curves and body fat composition for four groups. HA group: high-fat diet (HFD) feeding mice with *ad libitum*; dihydrotestosterone (DHEA) and *ad libitum* feeding (DA) group: mice were given an HFD and water *ad libitum*; HFD and TRF intervention (HT) group: mice were given an HFD as well as a TRF dietary pattern; DHEA and TRF intervention (DT) group: mice were given an HFD as well as a TRF dietary pattern. **(A)** Growth curves for four groups. **(B, C)** Body fat test. Values are mean ± SEM. *P<0.05, **P < 0.01, ***P< 0.001, ****P<0.0001, DA group vs. DT group; ^###^P<0.001, ^####^P<0.0001, HA group vs. HT group.

All mice were weighed weekly. There was a significant difference in body weight between the TRF and non-TRF groups, which increased progressively over time (**Figure 2A**). For body composition, fat mass rates were much lower in the mice treated with TRF compared to the control group (**Figure 2B**). As for lean body mass, an increase could be observed in the TRF groups (**Figure 2C**).

### 3.2 TRF improves glucose intolerance and reverses insulin resistance in mice with PCOS

**Figure 3:**
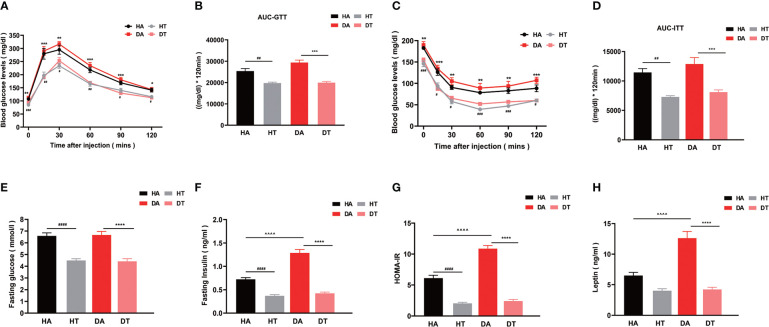
TRF improves glucose intolerance and reverses insulin resistance in mice with polycystic ovarian syndrome (PCOS). **(A, B)** Oral glucose tolerance test (OGTT, glucose 2 g/kg), **(C, D)** Insulin tolerance test (ITT, insulin 0.5 U/kg), **(E)** Plasma fasting glucose level in four groups. **(F)** Plasma fasting insulin level in four groups. **(G)** Homeostatic Model Assessment for Insulin Resistance (HOMA-IR), **(H)** Plasma leptin level. All data were presented as mean ± SEM **P < 0.01, ***P < 0.001, ****P < 0.0001, DA group vs. DT group; ^#^P < 0.05, ^##^P< 0.01, ^###^P < 0.001, ^####^P < 0.0001, HA group vs. HT group, ^^^^P < 0.0001, DA group vs. HA group.

To investigate the effect of changing dietary patterns on glucose homeostasis, we performed OGTT. Mice in the DT group exhibited significantly improved glucose intolerance in comparison to mice in the DA group (P < 0.001 at 12, 60, and 90 min; P < 0.01 at 30 min), as evidenced by lower blood glucose levels and a significantly lower AUC throughout (**Figures 3A, B**). The fasting glucose levels of mice in both HT and DT groups had a lower tendency compared to HA and DA groups (**Figure 3E**). Similar results existed between the HT group and the HA group, but the differences between the mice in the DT and HT groups were not significant (**Figures 3A, B, E**). ITT was used to further assess insulin sensitivity. Mice in both the HT group and the DT group showed higher insulin sensitivity compared with that in the non-TRF groups (**Figures 3C, D**). This improvement in glucose tolerance was also supported by fasting insulin levels (P < 0.0001, DT group vs. DA group; P < 0.0001, HT group vs. HA group) (**Figure 3F**). In addition, judged by Homeostatic Model Assessment for Insulin Resistance (HOMA-IR), the intuitive and most widely used quantitative index of IR, TRF significantly reversed IR in PCOS mice with an HFD or non-PCOS mice with an HFD (HT group vs. HA group, P<0.0001; DT group vs. DA group, P <0.0001; DA group vs. HA group, P<0.0001; respectively) (**Figure 3G**). Leptin is a product of the obesity gene and plays a role in the regulation of food intake, lipolysis, and glucose homeostasis (30). The plasma leptin level was also significantly lower in TRF groups, which further indicated that TRF could improve IR in PCOS mice (**Figure 3H**).

### 3.3 TRF ameliorates abnormal ovarian morphology in PCOS mice

**Figure 4:**
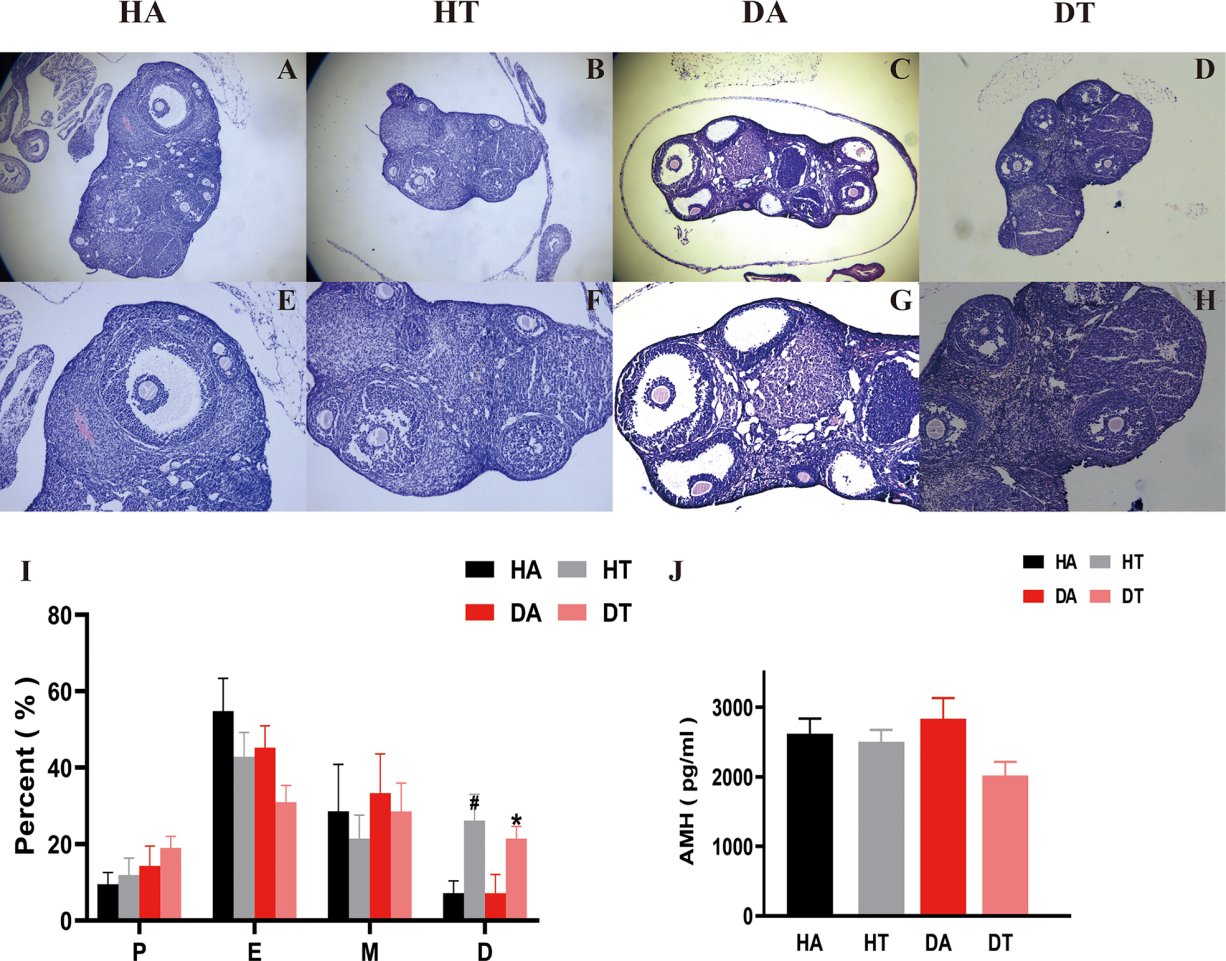
TRF ameliorates ovarian dysfunction in PCOS mice. **(A-H)** Hematoxylin and eosin **(H, E)** staining of typical sections of mouse ovary, Bar = 500 μm in A, B, C, and D. Bar = 250 μm in **(E–H)**. **(I)** Comparison of the percentage of estrous cycle (1 week) in each group 2 weeks after TRF. **(J)** Plasma anti-Müllerian hormone (AMH) level. *P<0.05, DT group vs. DA group. ^#^P<0.05, HA group vs. HT group. Data were presented as mean ± SEM.

Compared to the non-TRF group, mice in TRF groups showed a significant moderation in the duration of estrus and a gradual return to the normal cycle, i.e., a significant increase in the percentage of estrus and interestrus, which implies a remission of ovulatory disturbances (**Figure 4I**). This was further confirmed by ovarian morphology. Mice in the DA group exhibited typical ovulatory arresting ovarian polycystic changes: large, cavitary, and cystic-like follicles presented at the edge of the ovarian cortex and a reduced number of corpus luteum (**Figures 4C, G**). The ovaries of control mice treated with an HFD only behaved similarly but not as same as the DA group mice (**Figures 4A, E**). According to ovarian sections, mice treated with TRF had fewer cystic follicles and more corpus luteum compared to the non-TRF group (**Figures 4B, D, F, H**). AMH is one of the reliable biomarkers to evaluate ovarian function. Mice in the DT group had a lower level of AMH than that in the DA group, which further supports that TRF intervention could improve ovarian dysfunction in the PCOS mouse model (**Figure 4J**).

### 3.4 TRF ameliorates hyperandrogenemia in PCOS mice

**Figure 5:**
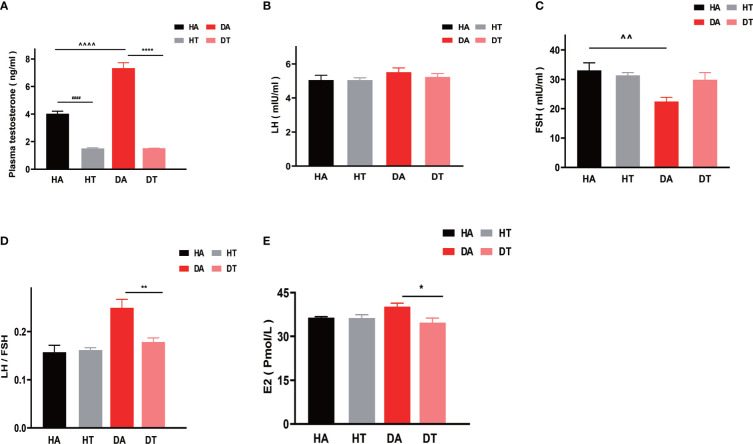
TRF ameliorates hyperandrogenemia in PCOS mice. **(A)** Plasma testosterone levels. **(B)** Plasma luteinizing hormone (LH) levels. **(C)** Plasma follicle-stimulating hormone (FSH) levels. **(D)**The ratio of LH to FSH. **(E)** Plasma estradiol (E2) levels. All data were presented as mean ± SEM. *P < 0.05, **P < 0.01, ****P < 0.0001, DA group vs. DT group; *P < 0.05, **P < 0.01, ****P < 0.0001, DA group vs. DT group; ^####^ P < 0.0001, HA group vs. HT group. ^P < 0.05, ^^P < 0.01, ^^^^P < 0.0001, DA group vs. HA group.

To further investigate the effect of TRF intervention on sex hormonal levels, especially on hyperandrogenemia, plasma testosterone, LH, FSH, and E2 levels were measured in the diestrus phase. Mice in the DT group and HT group had significantly reduced plasma testosterone levels (**Figure 5A**) compared to that in the DA group and HA group, respectively (DA group vs. DT group, P<0.0001; HA group vs. HT group, P<0.0001). Moreover, the plasma testosterone concentrations of mice in the DA group were higher than that in the HA group, indicating the successful establishment of the PCOS mouse model (DA group vs. HA group, P<0.0001) (**Figure 5A**). There was no difference in plasma LH levels between the groups (**Figure 5B**), but the ratio of LH to FSH (LH/FSH) was significantly different between the DA and DT groups (**Figure 5D**). FSH can promote follicular maturation. In the group treated with TRF, elevated FSH levels were consistent with improved ovarian morphology (**Figure 5C**). The plasma E2 concentrations of mice in the DT group were also different from those in the DA group (**Figure 5E**).

### 3.5 TRF-approved non-alcoholic fatty liver disease–like liver morphology in PCOS mice

**Figure 6:**
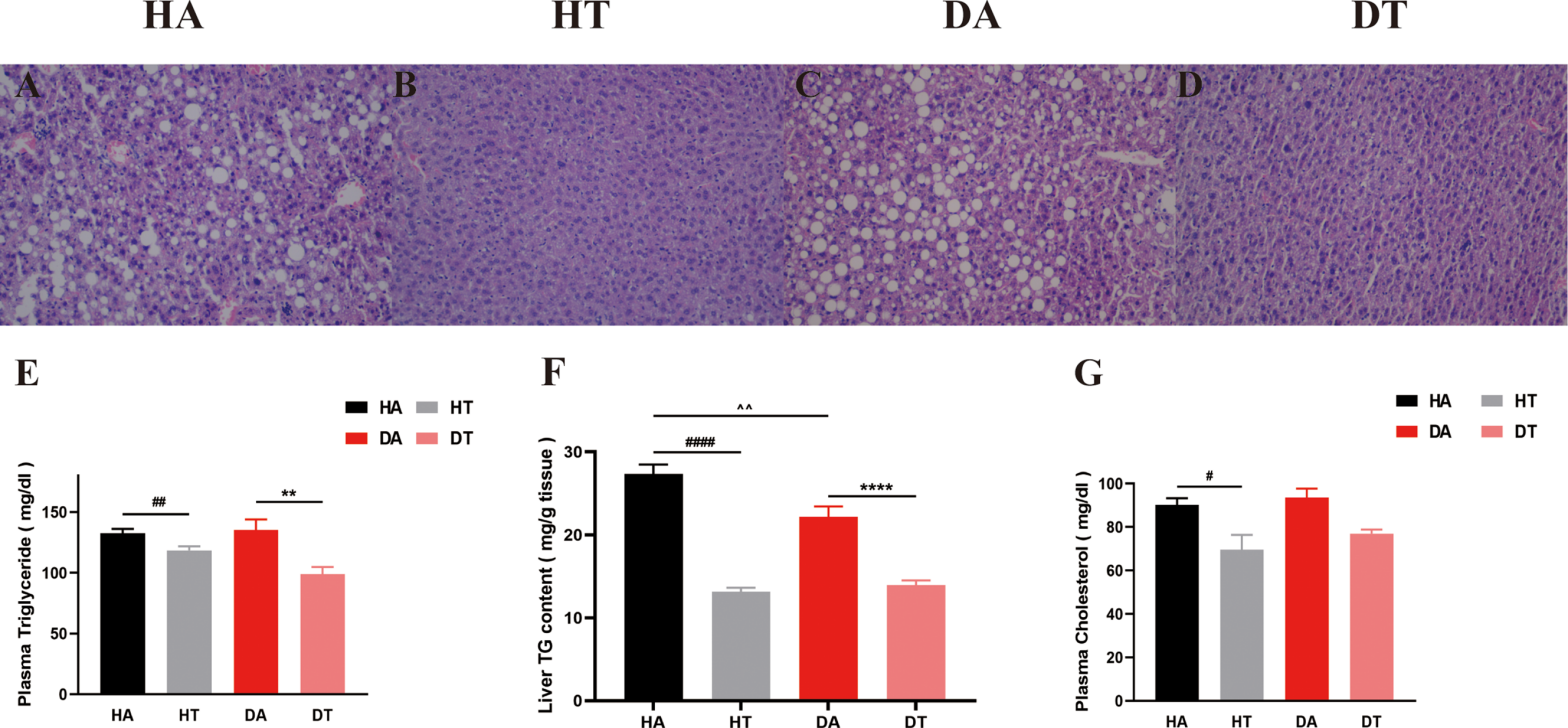
Representative liver morphology, triglyceride content in liver, and plasma lipid profiles in different groups. **(A-D)** H&E staining of liver sections (microscope whose magnification: ×20). **(E)** Triglyceride levels in plasma. **(F)** Triglyceride content in the liver. **(G)** Plasma cholesterol levels. All data were presented as mean ± SEM. **P < 0.01, ****P < 0.0001, DA group vs. DT group; ^#^P < 0.05, ^##^P < 0.01, ^####^P < 0.0001, HA group vs. HT group. ^^P < 0.01, DA group vs. HA group.

We further explored the effect of TRF intervention on lipid metabolism. As shown by H&E staining, liver lipid accumulation was severely increased in both HA and DA group mice (**Figures 6A, C**), which was ameliorated by TRF intervention in the HT and DT groups (**Figures 6B, D**). Liver triglyceride content, plasma cholesterol, and plasma triglyceride were tested using commercial ELISA kits. Plasma triglyceride levels and plasma cholesterol were significantly lower in TRF-treated mice than in *ad libitum*–fed mice (**Figures 6E, G**). A similar trend was observed for liver triglyceride levels (**Figure 6F**).

## 4 Discussion

Since TRF was proposed as a new model of intermittent food restriction, there have been many clinical and animal studies demonstrating its efficacy and safety. In this study, we showed that the DHEA combined with an HFD-induced PCOS mouse model had a significant typical PCOS phenotype, including an irregular estrus cycle, hyperandrogenism, polycystic ovarian morphology (PCOM), and metabolic syndrome manifestations such as obesity, IR, abnormal serum leptin level, impaired glucose tolerance, dyslipidemia, and non-alcoholic fatty liver disease (NAFLD). After 8 weeks of experimental intervention, TRF-treated mice had healthier glucolipid metabolism and significantly improved reproductive endocrinology compared to mice that ate *ad libitum*.

PCOS is a common female reproductive endocrine disorder, and the prevalence of obesity in PCOS patients varies from 30% to 76% according to many studies (31, 32), which is significantly higher than the normal population (33). Obesity exacerbates menstrual disorders and other clinical symptoms and increases the risk of T2DM, dyslipidemia, NAFLD, and cardiovascular diseases in PCOS patients (34). Consistent with the previous study that a time-limited diet improved weight of obese mice and decreased fat mass (16), our study showed that TRF could significantly reduce body weight and decrease fat mass in a PCOS mouse model.

IR is one of the important pathophysiological elements of PCOS, affecting nearly 65%–70% of women with PCOS (35). Although the high frequency of IR in PCOS patients was attributed to the high occurrence of obesity, subsequent clinical studies have found that PCOS patients with normal body weights and body masses also suffered from IR (36, 37). The inextricable relationship between hyperandrogenemia and IR is getting more attention as the understanding of this disorder increases (38, 39). Lifestyle interventions such as intermittent fasting can improve IR (40, 41). In the current study, we confirmed that obese PCOS mice also had IR and hyperinsulinemia, which was consistent with the early studies (42, 43). However, the IR of obese PCOS mice had been indigenously improved after 8 weeks of TRF intervention, which could be attributed to the restoration of circadian rhythm, the same mechanism of action as TRF in obese mice (16). Reduced gluconeogenesis capacity and increased tricarboxylic acid (TCA) and pentose phosphate pathways were also potential mechanisms of TRF intervention in IR (44–46).

Women with PCOS are often accompanied by lipid abnormalities (47). In our study, mice fed 8-h TRF at night showed a decreased level of both plasma triglycerides and total cholesterol in comparison with mice that had *ad libitum* access to food. Furthermore, from the liver morphology, many lipid droplets squeezed the normal hepatocytes of PCOS mice, disrupting the normal liver morphology and function. However, mice in the TRF group had improved liver morphology, showing fewer and smaller lipid droplets and an orderly organization. Meanwhile, the assay of triglyceride content in the liver showed that the triglyceride content in the liver of TRF mice was significantly decreased. All these confirmed that TRF ameliorates lipid distribution in the PCOS mouse model. There are some potential mechanisms. The effect of TRF on improving lipid profiles may be attributed to the combined action of biological circadian clock molecules and metabolic modulators. Previous studies have found that the expression of circadian clock genes *Reverb* and *Per2* was upregulated in the liver of TRF mice, which further inhibited downstream fatty acid synthesis and prolonged the related clock-controlled genes, resulting in a decrease in fatty acid synthesis (16, 48, 49). Our team will further explore whether TRF supports lipid homeostasis in PCOS mice through the same mechanism in the future.

Reproductive endocrine dysfunction is a prominent feature of PCOS, and hyperandrogenism is one of the most common features in women with PCOS (50, 51). Previous studies have suggested that abnormal secretion of ovarian theca cells may be an underlying cause of hyperandrogenism (52, 53). The plasma FSH level and LH level are considered to be clinical indices to assess ovarian reserve function and ovulation. A higher ratio of LH/FSH has a great diagnostic value for PCOS (54, 55). The PCOS mouse model in our study had typical hyperandrogenism and an elevated LH/FSH ratio. After 8 weeks of TRF treatment, plasma androgen levels and the LH/FSH ratio were significantly lower in PCOS mice, which was consistent with other dietary interventions to improve endocrine and metabolism in PCOS (56, 57). A previous study of a time-restricted diet on the reproductive system of female mice suggested that TRF may regulate gonadotropin-releasing hormone (GnRH) secretion through fibroblast growth factor 21 (FGF21) (21), which could further influence the synthesis of steroid hormones.

This is the first study to address the beneficial effect of TRF on the reproductive system, glucose metabolism, and lipid profiles in a PCOS mouse model. There were still several limitations in our study. Firstly, we did not explore the mechanism of TRF-induced reduction in plasma androgen levels. Secondly, in mice grouping, we did not add a low-fat feeding group to better mimic the differences in diet and body weight in clinical PCOS patients. In addition, we did not estimate the daily energy consumption of mice in each group. Finally, although all microscopic assessments were conducted by two independent accessors for double-checking, it still per se involves a degree of subjectivity. In the future, our team will further improve our experiments and explore relevant mechanisms.

## 5 Conclusion

As an emerging dietary treatment for obesity, TRF has a significant role in weight loss, which is more acceptable because of the unrestricted calorie intake for a defined period (58). In the present study, the data indicated that TRF significantly improved glycolipid metabolism, hyperandrogenemia, the menstrual cycle, and PCOM in obese PCOS mice, which provides new evidence for clinical lifestyle interventions in obese PCOS.

## Data availability statement

The original contributions presented in the study are included in the article/supplementary material. Further inquiries can be directed to the corresponding authors.

## Ethics statement

The animal study was reviewed and approved by The Institutional Animal Care and Use Committee of Xiamen University School of Medicine.

## Author contributions

The study concept and design were framed by CH and CL. YH, BL, WL, XW, WT, XT, HC, and CH performed the experiments and collected data. YH, BL, WL, and CL interpreted the results and contributed to the preparation, review, and revision of the manuscript. All authors contributed to the article and approved the submitted version.
